# Pre- and post-therapeutic evaluation of liver and spleen in type I and type III Gaucher’s disease using diffusion tensor imaging

**DOI:** 10.1007/s00261-022-03602-5

**Published:** 2022-07-22

**Authors:** Eman Alnaghy, Ahmed Abdel Razek, Ebrahim Abdelhalim

**Affiliations:** 1 Department of Diagnostic Radiology, Mansoura Faculty of Medicine, Mansoura University, Mansoura, Egypt; 2 Department of General Surgery, Faculty of Medicine, Horus University, Damietta, Egypt

**Keywords:** Gaucher’s disease, M.R. imaging, Diffusion tensor imaging, Liver, Spleen

## Abstract

**Purpose:**

To assess the role of diffusion tensor imaging in assessing liver and splenic parenchymal infiltration in Gaucher’s disease (G.D.) type I and III before and after therapy.

**Methods:**

A prospective study was conducted upon 28 consecutive patients with G.D. type I and III and 28 age and sex-matched controls. They underwent an MRI and DTI of the liver and spleen. Mean diffusivity (M.D.) and fractional anisotropy (F.A.) values of the liver and spleen were evaluated before and after treatment and compared with control.

**Results:**

There was a statistically significant difference in the M.D. value of the liver and spleen between untreated patients and controls and between control and treated patients and in the M.D. value of the liver and spleen between untreated and treated patients. There is a statistically significant difference in the F.A. value of the liver and spleen between untreated patients and controls and in the F.A. value of the liver and spleen between untreated and treated patients. Hemoglobin level was positively correlated with the M.D. value of the spleen. Clinical score was negatively correlated with M.D. value of the spleen and was positively correlated with F.A. values of the liver and F.A. values of the spleen. Spleen volume was negatively correlated with M.D. values of the spleen.

**Conclusion:**

Significant difference in M.D. and F.A. values of liver and splenic parenchyma in p

atients with type I and III G.D. and controls, and between untreated and treated patients. The M.D. and F.A. values were well correlated with some biomarkers of disease activity.

## Introduction

Gaucher’s disease (G.D.) is one of the most common storage diseases with an autosomal recessive inheritance pattern. It is a familial lipodystrophic condition caused by a deficiency in the enzyme glucocerebrosidase (G Case), leading to intracellular accumulation of glycosphingolipids in monocyte-macrophage systemic cells (Gaucher cells) inside the liver, spleen, and bone marrow. Hepatosplenomegaly and bone marrow infiltration with skeletal system affection are the most common presentations [1–3].

G.D. is divided into three types: type 1 (non-neuronopathic disease) having one mutation at least with visceral and skeletal affection mainly, which is protective of neurological involvement. Type 2 (neuronopathic, infantile) has various genotypes with severe mutations and is characterized by the progression of neurological symptoms and signs until death occurs, typically before the age of 4 years. Type 3 (juvenile) is a sub-acute type with a slow progression of neurological manifestations with variable degrees of visceral involvement [4–6].

Gaucher cells infiltrate organs, leading to low-grade inflammatory changes; the main symptoms and signs are hepatosplenomegalies, anemia, bone osteonecrosis, deformities and pain, and neurological deficits in patients with type II and III. Visceral involvement leads to focal hepatic fibrosis, steatosis, hemosiderosis, cirrhosis, and hepatocellular carcinoma (HCC). Early detection of splenic and hepatic infiltration is essential to start treatment. Therapy is available in two modalities: enzyme replacement therapy (ERT) and substrate reduction therapy (SRT) [6, 7].

Laboratory investigations and therapeutic monitoring of disease progression and response to ERT are done using markers such as hemoglobin concentration and platelet count and enzyme assays, such as β-glucosidase and chitotriosidase [8, 9].

### Genotyping

The most prevalent worldwide disease genotype is L444P. Other mutation forms include N370S, 84GG, and IVS2+1G. Homozygosis for L444P results in neuronopathic disease, while the presence of a single mutant N370S allele prevents neurological affection [7, 10–13]. Patients who are homozygous for the N370S have milder disease than patients with compound heterozygous.

The International Collaborative Gaucher Group (ICGG) has recently recommended multiplanar M.R. imaging for G.D. to calculate liver and splenic volume every 12 or 24 months. MRI can assess the pre-and post-therapy response of patients as well as provide functional assessment using diffusion-weighted MRI (DWI) and magnetic resonance spectroscopy (MRS) for evaluation of diffuse and focal hepatic lesions, bone marrow infiltration, and central nervous system affection. Other non-invasive methods for imaging G.D. include the U.S. for regular follow-up and elastography (Fibro Scan) to evaluate liver fibrosis [14].

Diffusion tensor imaging (DTI) detects the micromovement of water molecules and provides quantitative information regarding the magnitude and directionality of water diffusion in three-dimensional space. DTI can characterize the diffusion process's orientation variability, allowing assessment of diffusion directionality or anisotropy. DTI uses different metrics to distinguish between different tissue compartments. The most common metrics used are fractional anisotropy (F.A.) and mean diffusivity (M.D.). F.A. indicates the degree of diffusion directionality within a voxel; M.D. corresponds to the directional magnitude of water diffusion [15].

There is no previous publication on DTI assessment of liver and spleen in children with G.D. We tried to perform a non-invasive abdominal MRI technique that can assess the pre-and post-therapy response of G.D. patients by detecting the liver and splenic parenchymal infiltration burden.

### Aim of the work

To assess the role of DTI in quantitative assessment of the liver and splenic parenchymal infiltration in patients with G.D. type I and III before and after therapy.

## Material and methods

**Fig. 1:**
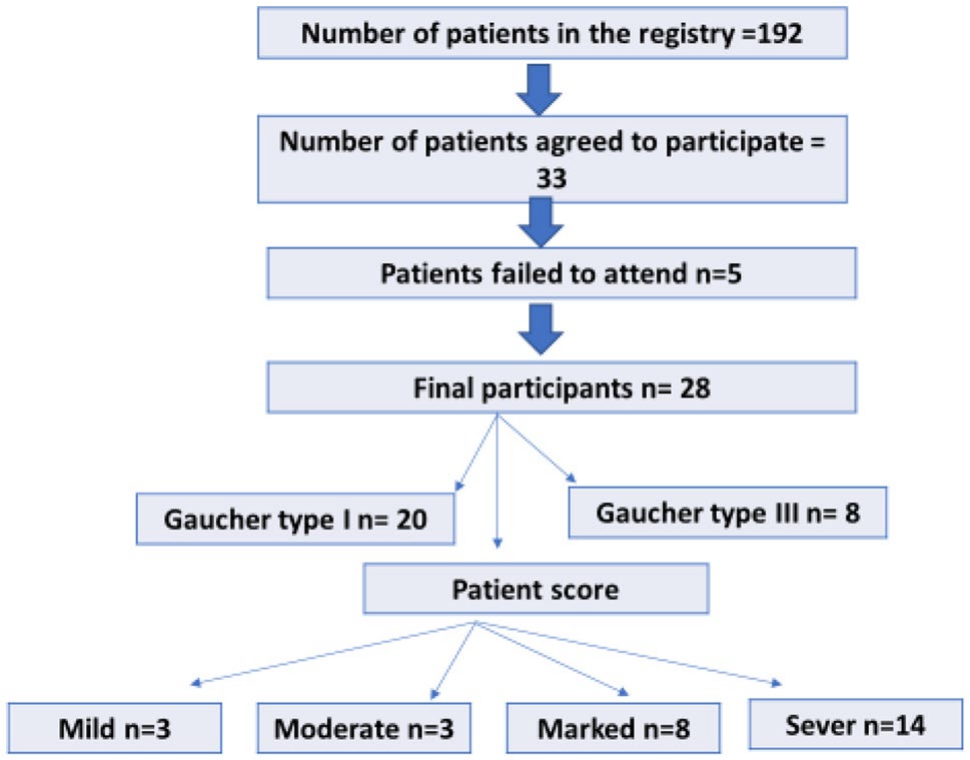
Patients flow chart

This is a prospective study that included 28 consecutive patients with type I and III GD [15 boys, 13 girls, mean age 6 years] and 28 controls (12 boys, 16 girls, mean age 7 years) with matched age and sex who performed MRI for other purposes (Table 1) and (Fig. 1). Institutional review board approval was obtained, and informed consent was obtained. Inclusion criteria included untreated patients with G.D. and diagnosed with low glucocerebrosidase levels. All patients and controls underwent a non-contrast MRI of the liver and spleen with a DTI assessment. The study was repeated for the patient group after therapy was completed. Genotyping was performed for all patients for the detection of known mutations. Results were correlated with the disease severity scoring system and some laboratory parameters, including Beta glucosidase, Chitotriosidase, hemoglobin (H.B.), and platelet levels assessed for patients and control.

**Table 1:** Baseline demographic and clinical characteristics of participants

	Patients	Control	Test of significance
	*n* = 28	*n* = 28	
Sex			
Males	15 (53.6%)	12 (42.9%)	*χ*^2^ = 0.644
Females	13 (46.4%)	16 (57.1%)	*p* = 0.422
Age/years			*t* = 0.810
Mean ± SD	6.2 ± 1.5	6.5 ± 1.26	*p* = 0.421
GD			
Type I	20 (71.4%)		
Type III	8 (28.6%)		
Score			
Mild	3 (10.7%)		
Moderate	3 (10.7%)		
Marked	8 (28.6%)		
Severe	14 (50%)

### Clinical scoring and volumetric assessment

A disease severity scoring system (DS3) allows the assessment of disease burden in patients [16, 17]. Patients were classified according to the score into Mild disease: (0–3), moderate disease: (3–6), marked disease: (6–9), and severe disease: score of more than 9. The liver and spleen volume was calculated using this formula: (0.524 × *W* × *T* × *L*) *W* is the maximum width, *T* is the thickness, and *L* is the length. The length of the liver and spleen was measured from superior to inferior; the width was measured from medial to lateral, and thickness from anterior to posterior [18].

*Laboratory parameters* Laboratory assessment of Beta glucosidase, Chitotriosidase, hemoglobin, and platelets were done for patients and control.

### M.R. imaging

The MR examinations were done using a 1.5 Tesla scanner (Ingenia, Philips). Routine MRI sequences were obtained first; axial T1 weighted images (TR/TE = 500/20 ms) and T2 weighted images (TR/TE = 4000/120 ms) of the abdomen were obtained. The DTI was also done with a single-shot echo-planar sequence (TR/TE = 3118/93 ms) with SENSitivity Encoding (SENSE). Then diffusion gradients were applied along 32 axes, using a b-value of (0 and 1000 s/mm^2^). FOV = 24–28 cm and data matrix = 92 × 88 were used, leading to voxel dimensions (2.43 × 2.54 × 2.5 mm). Slice thickness of 2.5 mm, no gap, and the total scan time = 7–8 min.

### Image analysis

**Fig. 2:**
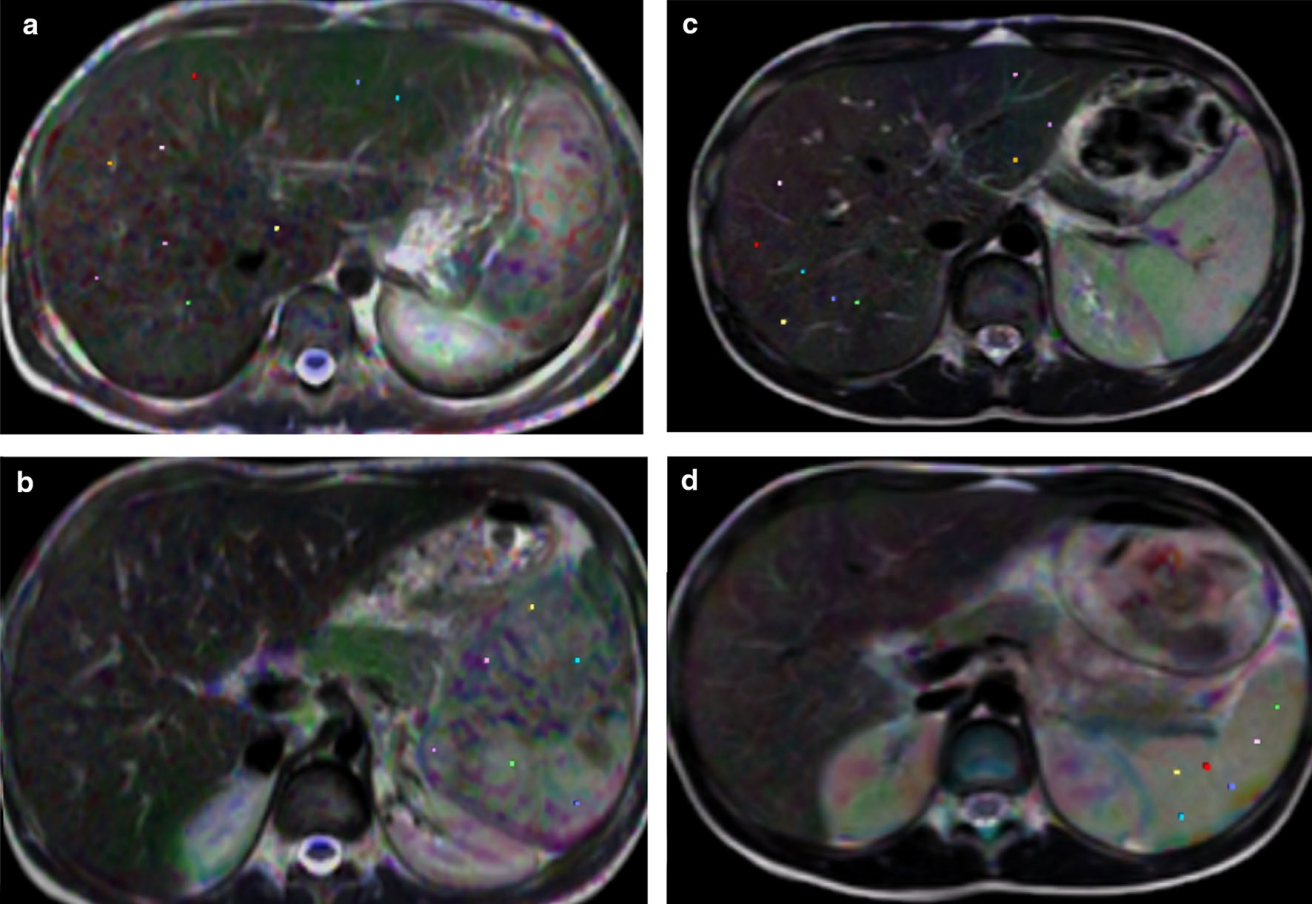
Showing ROI sits on DTI images of liver and spleen in patient (**a, b**), and in control (**c, d**)

Image analysis was done by one radiologist (E.A.), an expert in M.R. imaging for ten years who was blinded to clinical data. Images were transferred to a workstation (extended MR Workspace 2.6.3.5, Philips medical systems Nederland B.V.). Liver and spleen volumes were measured. In DTI processed images, 2–3 mm circular regions of interest (ROIs) were placed in the liver and spleen away from vessels and ribs, and measurement of M.D. and F.A. values of the liver and spleen in patient and control was done (Fig. 2). After measuring the average of 9 ROIs in the liver and 6 ROIs in the spleen, the final F.A. and M.D. values were obtained. ROIs were selected to be representative of liver and splenic parenchyma. In one previous study [15], final FA and MD values for each subject were the average of 6 ROIS placed in the lower three thoracic and upper three lumbar vertebral bodies away from the endplates.

### Statistical analysis

Statistical analysis was done using Statistical Package for Social Sciences (SPSS, Chicago, IL) version 22. Quantitative data were presented as mean and standard deviation (S.D.). The mean and standard deviation of the F.A. and M.D. of the selected regions in the patients (before and after therapy) and controls were calculated. Normally distributed data were compared between the three groups using independent samples *t* test. Data that violated the normality assumptions were compared using the Mann–Whitney test. Probability (*P*) values < 0.05 were considered statistically significant. The receiver operating characteristic (ROC) curve was done to evaluate the diagnostic capability of the F.A. and M.D. in differentiating patients from controls and patients before and after therapy with a calculation of area under the curve (AUC), accuracy, sensitivity, and specificity.

## Results

This prospective study included 28 patients with G.D. (15 boys, 13 girls, mean age of 6 years). Patients with G.D. type I (*n* = 20) and type III (*n* = 8), 28 matched controls (12 boys, 16 girls, mean age of seven years). Patients were classified according to the score into Mild disease (*n* = 3), moderate disease (*n* = 3), marked disease (*n* = 8) and severe disease (*n* = 14). Data analysis revealed the following results:

### M.D. values (Figs. 3a and 4a)

There was a statistically significant difference in the M.D. value of the liver and spleen between untreated patients and controls (*p* < 0.001) and between control and treated children (Table 2). Statistically, a significant difference was also detected in the M.D. value of the liver and spleen between untreated and treated patients (*p* < 0.001). The mean M.D. value of the liver and spleen in untreated children with Gaucher's disease was 1.31 ± 0.06 and 0.73 ± 0.09 × 10^−3^ mm^2^/s, respectively, in control children, it was 1.48 ± 0.07 and 0.98 ± 0.06 × 10^−3^ mm^2^/s, and intreated children it was 1.4 ± 0.04 and 0.86 ± 0.02 × 10^−3^ mm^2^/s, respectively. At the ROC curve, the AUC of the M.D. values of the liver and spleen used to differentiate untreated patients from controls was 0.96 and 0.98, respectively, with liver and spleen cutoff points to differentiate both groups being 1.4 and 0.90 × 10^−3^ mm^2^/s. respectively. Sensitivity, specificity, positive, and negative predictive values for liver and spleen were (96%, 84.6%, 92.3%, and 91.7%) and (92%, 92.3%, 95.8%, and 85.7%) with 92% accuracy for both. At the ROC curve, the AUC of the M.D. values used to differentiate untreated from treated patients was 0.9 and 0.86 for the liver and spleen, respectively, with liver and spleen cutoff points to differentiate both groups being 1.37 and 0.78 × 10^−3^ mm^2^/s, respectively. Sensitivity, specificity, positive and negative predictive values for liver and spleen were (84%, 84%, 84%, and 84%) and (92%, 92%, 76.7%, and 0.9%) with 84% and 82% accuracy, respectively.

**Fig. 3:**
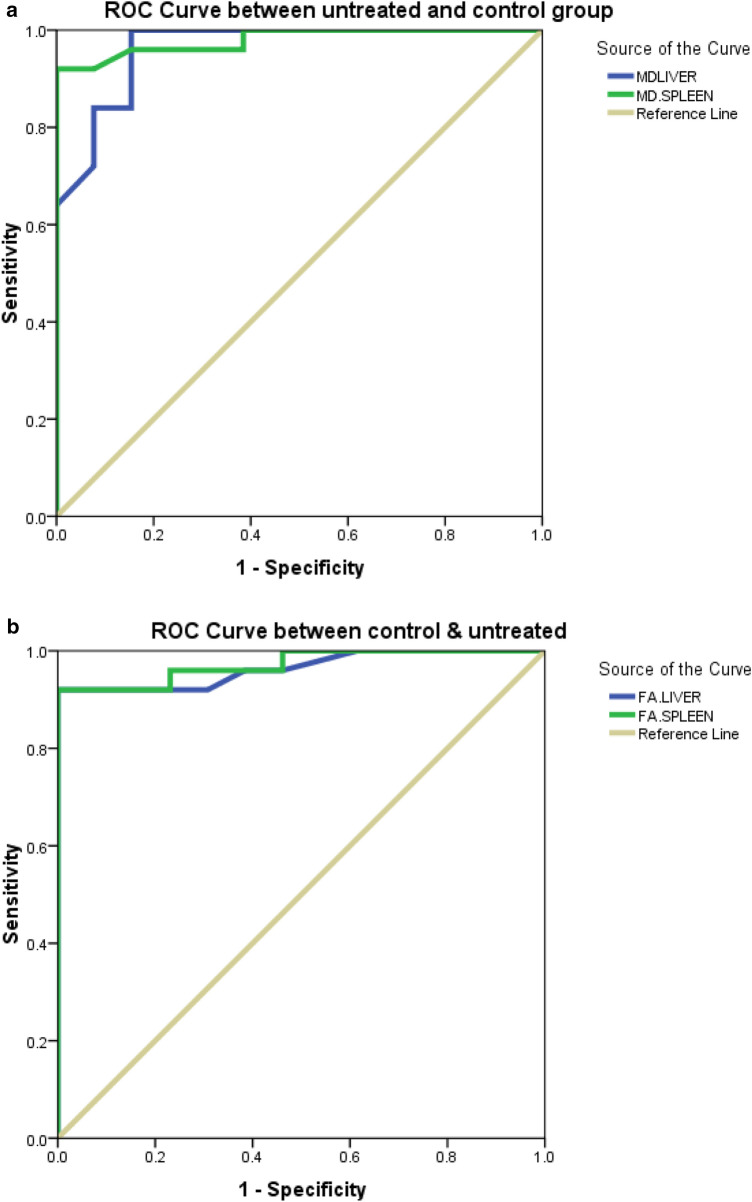
**a** Receiver operating characteristic (ROC) curve for M.D. values of the liver and spleen in untreated patients and control. **b** Receiver operating characteristic (ROC) curve for FA values of the liver and spleen in control and untreated patients

**Fig. 4:**
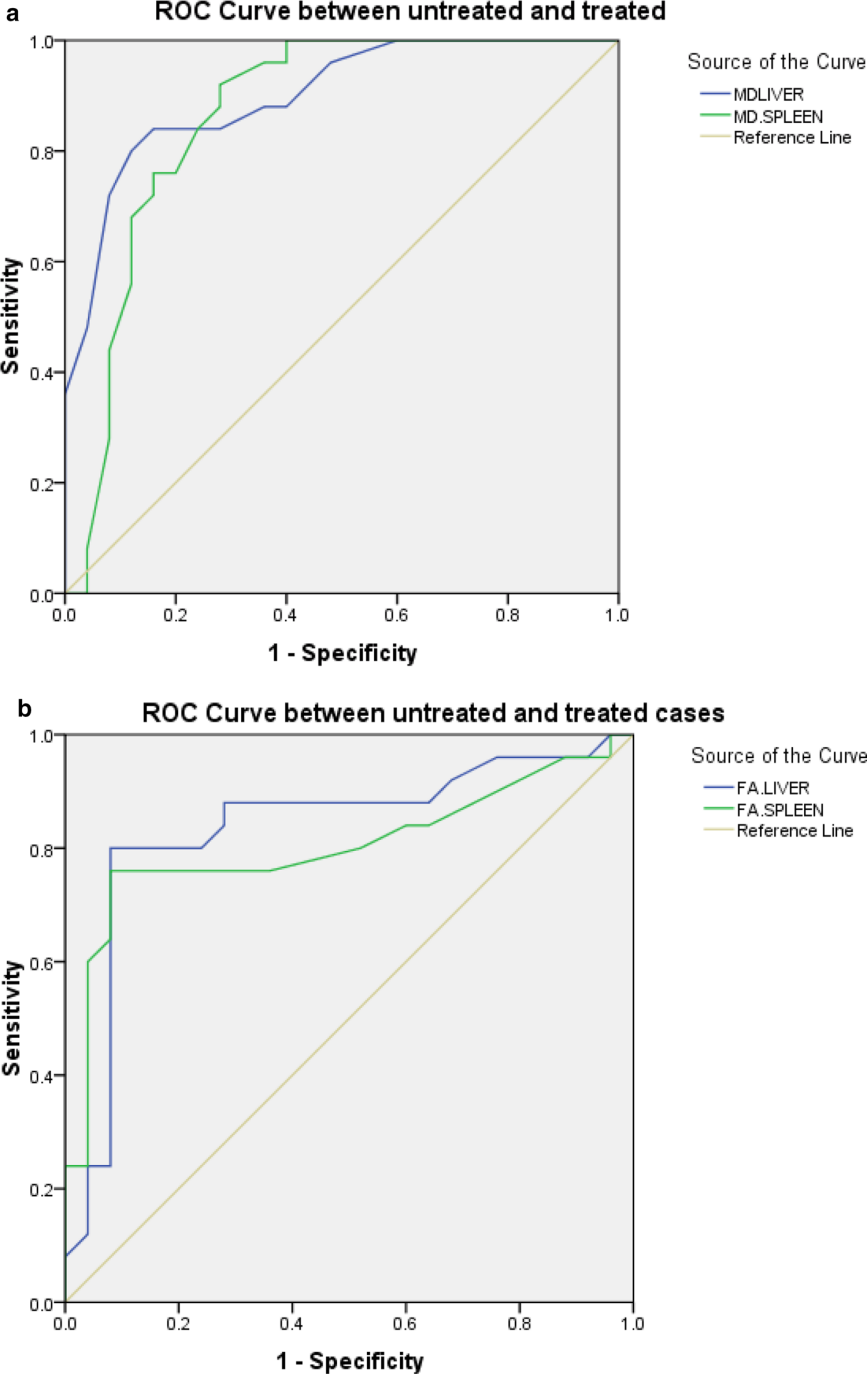
**a** ROC curve for M.D. values of the liver and spleen in untreated and treated patients. **b** ROC curve for F.A. values of the liver and spleen in untreated and treated patients

**Table 2:** Mean, median, SD, minimum, maximum, *p* value, of M.D. and F.A. of liver and spleen of untreated, treated patients and controls

	Liver MD	Liver FA	Spleen MD	Spleen FA
Controls * n* = 13	1.48 ± 0.07 ^ab^	0.29 ± 0.04 ^ab^	0.98 ± 0.06 ^ab^	0.28 ± 0.08 ^ab^
Untreated patients	1.31 ± 0.06 ^ac^	0.49 ± 0.08 ^ac^	0.73 ± 0.09 ^ac^	0.52 ± 0.08 ^ac^
Treated patients	1.4 ± 0.04 ^bc^	0.37 ± 0.08 ^bc^	0.86 ± 0.04 ^bc^	0.38 ± 0.12 ^bc^
One way ANOVA test	*p* = 42.82	*F* = 31.89	*F *= 47.73	*F *= 26.04
	*p* < 0.001*	*p* = 0.002*	*p* < 0.001*	*p* = 0.001*

Similar superscripted letters denote significant difference between groups by post HOC Tukey test

### F.A. values (Figs. 3b and 4b)

There was a statistically significant difference in the F.A. value of the liver and spleen between untreated patients and controls and between control and treated children (*p* = 0.002 and 0.001, respectively). Statistically, a significant difference was also detected in the F.A. value of the liver and spleen between untreated and treated patients (*p* = 0.02 and 0.03, respectively). The mean F.A. value of the liver and spleen in untreated children with G.D. was 0.49 ± 0.08 and 0.52 ± 0.08; in control children, it was 0.29 ± 0.04 and 0.28 ± 0.08, and intreated children it was 0.37 ± 0.85 and 0.38 ± 0.1, respectively. At the ROC curve, the AUC of the F.A. values of the liver and spleen used to differentiate untreated patients from controls were 0.96 and 0.97, with 0.33 and 0.38 cutoff points to differentiate both groups for the liver and spleen, respectively. Sensitivity, specificity, positive and negative predictive values for the liver and spleen were (92%, 69.2%, 85.2%, and 81.8%) and (92%, 76.9%, 88.5%, and 83.3%) with 84.2% and 86.8% accuracy, respectively. At the ROC curve (Table 3), the AUC of the F.A. values of the liver and spleen used to differentiate untreated from treated patients were 0.83 and 0.8, respectively, with a cutoff point to differentiate both groups for the liver and spleen of 0.41 and 0.4, respectively. Sensitivity, specificity, positive and negative predictive values for the liver and spleen were (80%, 92%, 90.9%, and 82.1%) and (76%, 92%, 90.5%, and 79.3%) with 86% and 84% accuracy, respectively.

**Table 3:** The ROC curve results of MD and FA of patients vs controls and untreated vs treated

	AUC	Cut off point	Sensitivity	Specificity	PPV	NPV	Accuracy
Untreated vs controls							
MD liver	0.96 (0.906–1.0)	1.4	96	84.6	92.3	91.7	92.1
FA liver	0.96 (0.911–1.0)	0.33	92	69.2	85.2	81.8	84.2
MD spleen	0.98 (0.944–1.0)	0.9	92	92.3	95.8	85.7	92.1
FA spleen	0.97 (0.927–1.0)	0.38	92	76.9	88.5	83.3	86.8
Untreated vs treated							
MD liver	0.90 (0.817–0.983)	1.37	84	84	84	84	84
FA liver	0.83 (0.711–0.959)	0.41	80	92	90.9	82	86
MD spleen	0.86 (.756–0.975)	0.78	92	92	76.7	90	82
FA spleen	0.80 (0.672–0.936)	0.4	76	92	90.5	79.3	84

### Clinical scoring

According to the clinical score, patients were classified into Mild disease (*n* = 3), moderate disease (*n* = 3), marked disease (*n* = 8) and severe disease (*n* = 14).

*Laboratory* (Table 4) Blood levels of Beta glucosidase, hemoglobin, and platelets were significantly lower in patients with G.D. than in control; Beta glucosidase level was (0.54 ± 0.15 and 3.64 ± 0.6, *p* < 0.001) for patients and control, respectively. Hemoglobin level was (7.73 ± 0.1 and 11.03 ± 0.6, *p* < 0.001) for patients and control, respectively. Platelets level was (92.12 ± 06.4 and 207.62 ± 36, *p* < 0.001) for patients and control, respectively. The chitotriosidase level was significantly higher in patients with G.D. than in control; (5044 ± 2949 and 1673 ± 130, *p* < 0.001) for patients and control.

**Table 4:** Mean and SD, min and max of lab: beta glycosidase, chitotriosidase, HB, Platelets Beta glucosidase, Clin: DS3 score, Vol: liver and spleen

	Control	Cases (untreated)	Test of significance
Laboratory			
Beta glucosidase	3.64 ± 0.64 (2.7–4.7)	0.54 ± 0.15 (0.21–0.85)	*t* = 23.6, * p* < 0.001*
Chitotriosidase	1673.77 ± 130.12 (1509–1872)	5044.38 ± 2949.37 (1124–14,576)	*t* = 4.09, * p* < 0.001*
HB	11.03 ± 0.60 (10.2–12.3)	7.73 ± 1.01 (5.7–9.0)	*t* = 10.752, * p* < 0.001*
Platelets	207.62 ± 36.11 (158–278)	92.12 ± 6.42 (79–104)	*t* = 15.71, * p* < 0.001*
Clinical			
DS3	NA	10.28 ± 3.38 (5–19)	
Volume	NA		
Liver		671.60 ± 132.79 (488–955)	
Spleen		387.32 ± 150.28 (145–666)	

*significant *p* value

*Liver and spleen volume* The mean liver volume in patients with G.D. was 671.6 ± 132, and the mean splenic volume was 387.32 ± 150.

*Genotyping* There was no statistically significant difference in the M.D. values of the liver between untreated patients with L444P mutation (*n* = 11) and patients with other mutations (*n* = 17). M.D. liver values were (1.31 × 10^−3^ mm^2^/s and 1.3 ± 0.05 × 10^−3^ mm^2^/s, *p* = 0.7) for patients with L444P mutation and patients with other mutations, respectively. Spleen MD values were (0.78 ± 0.01 and 0.7 ± 0.08, *p* = 0.05), respectively. FA values of the liver were (0.48 ± 0.08 and 0.5, *p* = 0.4), spleen F.A. values were (0.5 ± 0.09 and 0.53, *p* = 0.5) for patients with L444P mutation and patients with other mutations, respectively.

*Correlations* Chitotriosidase level was positively correlated with FA values of the liver (*r* = 0.409, *p* = 0.042) and spleen (*r* = 0.39, *p* = 0.05) in untreated patients. Hemoglobin level was positively correlated with the M.D. value of the spleen (*r* = 0.5, *p* = 0.01) and negatively correlated with F.A. values of the spleen (*r* = 0.46, *p* = 0.02). Clinical score was negatively correlated with the M.D. value of the spleen (*r* = 0. 67, *p* < 0.001) and was positively correlated with F.A. values of the liver (*r* = 0.5, *p* = 0.01) and F.A. values of the spleen (*r* = 0.41, *p* = 0.02). Spleen volume was negatively correlated with M.D. values of the spleen (*r* = 0. 40, *p* = 0.047).

## Discussion

In this study, the M.D. value of liver and spleen in untreated patients with G.D. was significantly lower than in controls. This was attributed to Gaucher cell accumulation in the liver and splenic parenchyma with subsequently increased cellularity and restricted diffusion. M.D. value is inversely related to tissue cellularity resulting in lower diffusivity of the liver, and splenic Gaucher cells infiltrated parenchyma. This agrees with previous studies that reported that brain and bone marrow Gaucher cells infiltrated parenchyma show restricted diffusion with low ADC values [14, 15, 18–22].

There was also a significant difference in the M.D. value of the liver and spleen between untreated and treated patients; treated patients showed higher M.D. values than untreated as the burden of infiltration by Gaucher cells is higher than treated patients. Decreased hepatic and splenic parenchyma cellularity increases diffusivity of the liver and spleen in treated patients. On the contrary F.A. values of liver and spleen in untreated patients were significantly higher than that of controls and also with significant difference detected in F.A. values between controls and treated patients.

### Clinical scoring

A validated DS3 for G.D. guides clinicians to start specific therapy monitors disease progression and response to therapy and compares different patients in clinical studies [16, 17]. The DS3 could assess patient condition, classify patients into subgroups, and compare outcomes among patients. In our study, the clinical score was positively correlated with F.A. values of the liver and spleen and negatively correlated with the M.D. value of the spleen, indicating that the more decrease in the M.D. value and the more increased F.A. values, the higher will be the patient score.

### Volumetric and hematological assessment

In the treated patient with ERT and SRT, treatment is targeted to macrophages, increasing the breakdown of the accumulated glycolipids; this decreases the burden of accumulation inside the visceral parenchyma. SRT inhibits glucosylceramide synthesis, decreasing its amount and leading to less visceral and hematological complications. Visceral (decreased volume of liver and spleen) and hematological improvement (cytopenia) are apparent after six months of treatment [21, 23].

In our study, spleen volume was negatively correlated with M.D. values of the spleen, indicating that the more splenomegaly, the *more parenchymal affection* with decreased M.D. values. Also, H.B. level was positively correlated with the M.D. value of the spleen, indicating that laboratory improvement with increased hemoglobin level will be accompanied by decreased burden of Gaucher cells accumulation and increased spleen M.D. value.

In our study, Chitotriosidase level was positively correlated with F.A. values of the liver and spleen, indicating that the more increased level of Chitotriosidase, the higher liver F.A. values will be detected. According to our study, the addition of DTI to routine M.R. imaging protocol has been of great value as a quantitative parameter to detect hepatic and splenic parenchymal infiltration in children and monitor response to treatment.

The limitations of this study are due to the small number of patients as the disease is not so common. Further multicenter studies with many patients are recommended with an evaluation of the patient's prognosis.

## Conclusion

We concluded a significant difference in the M.D. and F.A. values of liver and spleen between children with G.D. and controls, also before and after therapy. The MD and F.A. values were well correlated with some biomarkers of disease activity.

## Abbreviations

G.D.: Gaucher’s disease
DTI: Diffusion tensor imaging
MD: Mean diffusivity
F.A.: Fractional anisotropy
MRI: Magnetic resonance imaging
ERT: Enzyme replacement therapy
SRT: Substrate reduction therapy
ROC: Receiver operating characteristic
AUC: Area under the curve
